# Improving Outcomes in Urological Surgery for the Elderly: Strategies for Optimization and Risk Reduction

**DOI:** 10.1007/s11934-025-01284-2

**Published:** 2025-06-30

**Authors:** Patrick Juliebø-Jones, Christian Beisland

**Affiliations:** 1 Department of Urology, Haukeland University Hospital, Bergen, Norway; 2 Department of Clinical Medicine, University of Bergen, Bergen, Norway; 3 EAU YAU Urolithiasis group, Bergen, Norway

**Keywords:** Elderly, Surgery, Complicartions, Safety, Mortality

## Abstract

**Purpose of Review:**

The purpose was to present latest findings on factors that can help improve risk profiling for surgery in the elderly and thus improve outcomes.

**Recent Findings:**

Approximately two thirds of patients undergoing urological surgery are elderly. A number of assessment tools are now available for clinical application to facilitate risk planning when considering surgery. There is an overall lack of trials performed in the elderly on account of a number of factors including cognitive impairment, mobility and perceived lack of benefit. Clinicians are generally poor at estimating 10-year survival in patients and usually underestimate it. Treatment success in this demographic varies from the index patient and an individualised approach should be taken.

**Summary:**

It is of increasing relevance for clinicians to familiarize themselves with tools that can facilitate surgical care in the elderly. Prospective studies are needed, which also monitor outcomes in patients who did not undergo surgery.

## Introduction

Life expectancies have risen worldwide and continue to do so. In the US, the number of persons aged over 65 years is expected to double between 2000 and 2050 [1]. Globally, it is projected that the number in this age demographic will surpass the number of children under 18 years by this time point also [2]. Declining birth rates in many countries have also had a resultant effect on increasing the proportion of older persons. In societies where the proportion of persons > 65 years exceeds 14%, they are no longer considered as *ageing* but rather as *aged* (e.g. United States, Norway). Indeed, beyond 21%, they are termed *super-aged* (e.g., Japan, Italy). Life expectancies are also increasing rapidly in low- and middle-income countries (LMICs) [3].

While improved life expectancies should be celebrated as a landmark for advances in medical care, health care is more complex in this demographic. It is also considered a core contributor to the rising proportion of health expenditure as a percentage of GDP [4]. The need for health systems to adapt to these changes has been set as a global priority according to the World Health Organisation (WHO) and the focus aligns with the United Nations Decade of Healthy Ageing (2021–2030). In a report from the UK National Confidential Enquiry into Patient Outcome and Death (NCEPOD), the committee determined that healthcare provided to the elderly was insufficient and stressed that a “one size fits all medicine model” cannot be applied to this heteregenous population [5].

It has been reported that at least two thirds of urological surgeries are now performed in elderly patients [6]. The risk of post operative complications is widely documented to be higher in this group. “Age does not come alone” is a commonly cited adage and in this regard, accompanying factors include frailty, cognitive decline, and diminished physiological reserves. These are considered to contribute more significantly to a patient’s surgical morbidity profile than their chronological age alone. Navigating this complex interplay can therefore be a challenge for clinicians and yet the demand for planning urological surgery in patients belonging to this special population is going to be a more common clinical encounter. To this end, awareness of strategies for optimising care and reducing associated risks is needed. Research and attention towards this problem has increased in recent years with the term *urogerontology* emerging as a result and national societies forming subspeciality groups. Our objective was to perform a review to outline and summarise this work in the field of urological surgery.

## Materials and Methods

A comprehensive literature review was performed of literature on the subject of surgery in older persons, with a focus on those performed in the field of urology. Bibliographic databases searched included (but not limited to) Pubmed/Medline, Google Scholar and the Cochrane Library. All article types in the English language were considered with no time restrictions. The data was reviewed and has been summarised in a narrative format.

## Results

### The Documented Complication Burden

Surgical complications are widely documented to occur more commonly in older persons (Table 1). This is accompanied by higher mortality rate, longer postoperative stays and a higher chance of being discharged to a facility other than their normal residence [7]. Complications could also be higher than what is reported in studies given emergency appointments with primary care or nursing home doctor may not be tracked in their hospital records at the time of study reporting [8]. In a review of complications post radical cystectomy (RC) in elderly patients, Froehner et al., reported that the difference in rates between age groups is even higher when comparing 30 days to 90 days [9]. In a more recent study addressing mortality rates post RC, 90-day mortality increased > 80 years (odds ratio [OR] 3.42, 95% confidence interval [CI] 1.62–7.22). Most urological studies focus on 30-day outcomes for reporting adverse events, while in this age group it is arguably important to extend this to 90 days to give greater insight. In a study of patients aged ≥ 85 years undergoing ureteroscopy for kidney stones, 41% suffered a complication of some kind within 90 days [10].

**Table 1:** Key themes and summarised findings

Theme	Summary
**Surgical complications**	Post operative complications, readmission rate and mortaliy are higher in elderly patients
**Predicting life expectancy**	Health professionals are poor at predicting 10-year survival. Prediction models and actuarial predicted survival data are superior but still lack precision
**Geriatric assessment tools**	Recommended tools include G8, Mini-Cog and CFS Limited use by urologists currently.
**Interdisciplinary care**	Implementation of geriatric liaison service has been associated with shorter length of stays and lower complication rates
**Considering a conservative approach**	The decision to adopt a conservative approach should be weighed up more carefully in this population.
**Treatment success**	What is considered treatment success should not necessarily be the same as for the younger index patient.
**Challenges for research**	Ethical concerns, travel, mobility and limited perception of study benefit include core reasons why research of this kind is lacking

### Predicting Life Expectancy

A core element in decision making in surgery for the elderly is their expected life expectancy, particularly over the next 10 years. The latter is for example, a common decision factor when considered radical prostatectomy (RP) for localised prostate cancer. A number of studies have consistently found that both doctors and nurses are poor at estimating 10-year survival and predominantly underestimate it [11, 12]. Accuracy for this particular dichotomous outcome is far more accurate on a short-term basis but the horizon effect occurs as increasing imprecision of predictions is observed as the time frame extends further into the future [12].

More objective tools include statistical models based on registry cohorts of populations with a particular disease such as the model described by Zhou et al. for predicting risk of death post radical prostatectomy using the Surveillance, Epidemiology, and End Results (SEER) database [13]. Another option for clinicians is use of actuarial predicted survival data sourced from national statistics and these are the most commonly used by institutions such as National Institute for Health and Care Excellence (NICE) when making recommendations for treatment considerations. A previous review evaluating all these different methods found that these tools are superior to clinican predicted life expectancy but still lack accuracy [12]. Using purely age cut offs when deciding suitability for treatment is discouraged, and is increasingly considered to constitute “ageism” [14]. Rather, individual patient characterics should should be weighed up while appreciating their wishes and projected life expectancy.

### Geriatric Assessment Methods

A number of tools are available to aid the evaluation of health status and that can provide more information beyond just age and performance status alone. Their regular clinical application receives a *strong* recommendation from the European Association of Urology (EAU) Prostate cancer guidelines [15]. The Geriatric 8 (G-8) screening tool is specifically highlighted. Developed by Ballera et al., it can be used to identify cancer patients who would benefit most from a comprehensive geriatric assessment (CGA) [16]. It includes seven items from the Mini Nutritional Assessment (MNA) questionnaire that cover nutrition, mobility, cognitive status, polypharmacy with the final component being the patient´s age. It is freely available in an online calculator format also. Possible scores range from 0 to 17 with lower values indicating poorer health status and the guidelines advise full assessment for < 15. The tool can be useful to identify potentially reversible conditions that can be improved before a final decision is made on the feasibility of surgery.

An alternative to the G-8, is the Erlangen Index (EI) that covers five areas of geriatric assessment including mobility, dependence status, frailty, American Society of Anesthesiologists (ASA) physical status and the Charlson Comorbidity Index (CCI) [17]. Designed for assessing elderly patients undergoing major uro-oncological surgery, Graf et al., recently validated this tool and determined it as a reliable method for identifying elderly patients not only at high risk of mortality but also persistent functional impairment [18].

Other tools to consider incorporating are those for specific aspects such as for frailty (e.g., Clinical Frailty Score (CFS)) and cognitive function (e.g., Mini-Cog). Sun et al. found that CFS scores ≥ 5 were associated with a more than 10-fold increase in the risk of major complications after uro-oncological surgery and over a 20-fold increase in the risk of functional decline at 90 days [19]. Eredics et al. found that frailty was the biggest predictor for one year mortality for nonagerians admitted acute to urology wards [20]. Yajima et al. reported that a Mini-Cog score of < 3 was a clear precictor (OR = 9.5; *p* < 0.001) for delirium after urological surgery [21]. Of note, however, a recent survey highlighted that less than 15% of urologists use geriatric assessment tools in their clinical practice [22]. Why this is so, is likely multifactorial. It may be partly attributable to lack of awareness. Practical challenges may also be a contributor too. More extensive geriatric assessments such as the GCA are time consuming and can represent a resource that is not available. The GCA itself, refers for several assessments made by wider members of the multidisplinary team and is a process rather than a single evaluation in time [23]. In a systematic review by Partridge et al., the authors found that implementation of GCA improves post-operative outcomes the elderly [24]. However, there is such heterogeneity in how they are performed, that the authors concluded that it remains unanswered which part of GCA actually contributes the most, the so called ¨*blackbox effect*´.

### Clinical Interventions

Braude et al., initiated a Proactive care of Older People undergoing Surgery (POPS) intervention for elderly inpatients in urology [25]. Through implememntation of regular multidiscipinlary meetings and ward round involvement from a geriatrician, the length of stay was decreased by 19% as well as fewer complications and re-admissions. This method of POPS has been previously applied across a range of specialities with good effect [26]. Hospital at home (HaH) is another initiative that has been promoted across several countries. It facilitates a step down from the ward setting where patient can still receive much of the care that is traditionally only deliverable in secondary care such as intravenous medication and monitoring. In a Spanish cohort study of 325 patients undergoing major surgeries including RC, it allowed for shorter length of stay and re-admission rate of 7% [27].

### Decision Making – Adopting a Conservative Approach

The maxim “Good surgeons know how to operate; better ones know when to operate; and the best know when *not* to " is well-established in surgical tradition—yet it is perhaps even more relevant when treating patients in this age group. While numerous studies do report acceptable outcomes in elderly patients, caution is warranted when attempting to generalise these findings. Due to the retrospective nature of these studies, there is an inherent selection bias: patients with significant comorbidities who underwent surgery were typically deemed suitable by experienced clinicians at tertiary centres, based on clinical judgments that may not be fully captured or reproducible within the study data [10, 28]. While it is still absolutely positive to have reports from tertiary centres that support the feasibility of such surgeries, what would be even more valuable would be for the follow up outcomes to be attained for those who were deemed inoperable.

There are a lack of studies exploring patient perspectives when facing the decision of surgery in the elderly. However, in a study by Lavery et al., older age was identified as significant predictor for decisional regret associated with RP [29]. García-Rodelas et al., reported findings from a qualitative study of elderly men who had undergone RP and reported consistently poor experiences among the participants [30]. Ensuring that patients are clearly informed about their alternatives, including the discussion around ‘What if we do nothing?‘, is a key aspect of the Scottish approach known as ‘Realistic Medicine’ [31]. This aligns closely with the principles of ‘Choosing Wisely’ in the United States [32]. It is therefore important for clinicians to ensure information has been communicated in an appropriate way and to make adjustments as necessaey including regarding health literacy [33].

### Treatment Success

In this age group, treatment success should arguably be defined more broadly. For example, in a young patient with renal stones, success may not be considered to have been achieved if follow up imaging does not show a 100% fragment free status [34]. However, in an elderly patient, dealing with the culprit stone is arguably the priority and the threshold to accept residual fragments can be higher. Residual fragments should not therefore constitute unsuccessful treatment. When performing bladder outflow surgery for an extremely elderly male with a long-term catheter, their predicted life expectancy may be limited. However, even one or two years living potentially catheter free may have such a positive impact on their quality-of-life treatment, that surgery should be considered even if the estimated life expectancy is relatively short.

### Challenges in Undertaking Research Studies in the Elderly

**Fig. 1:**
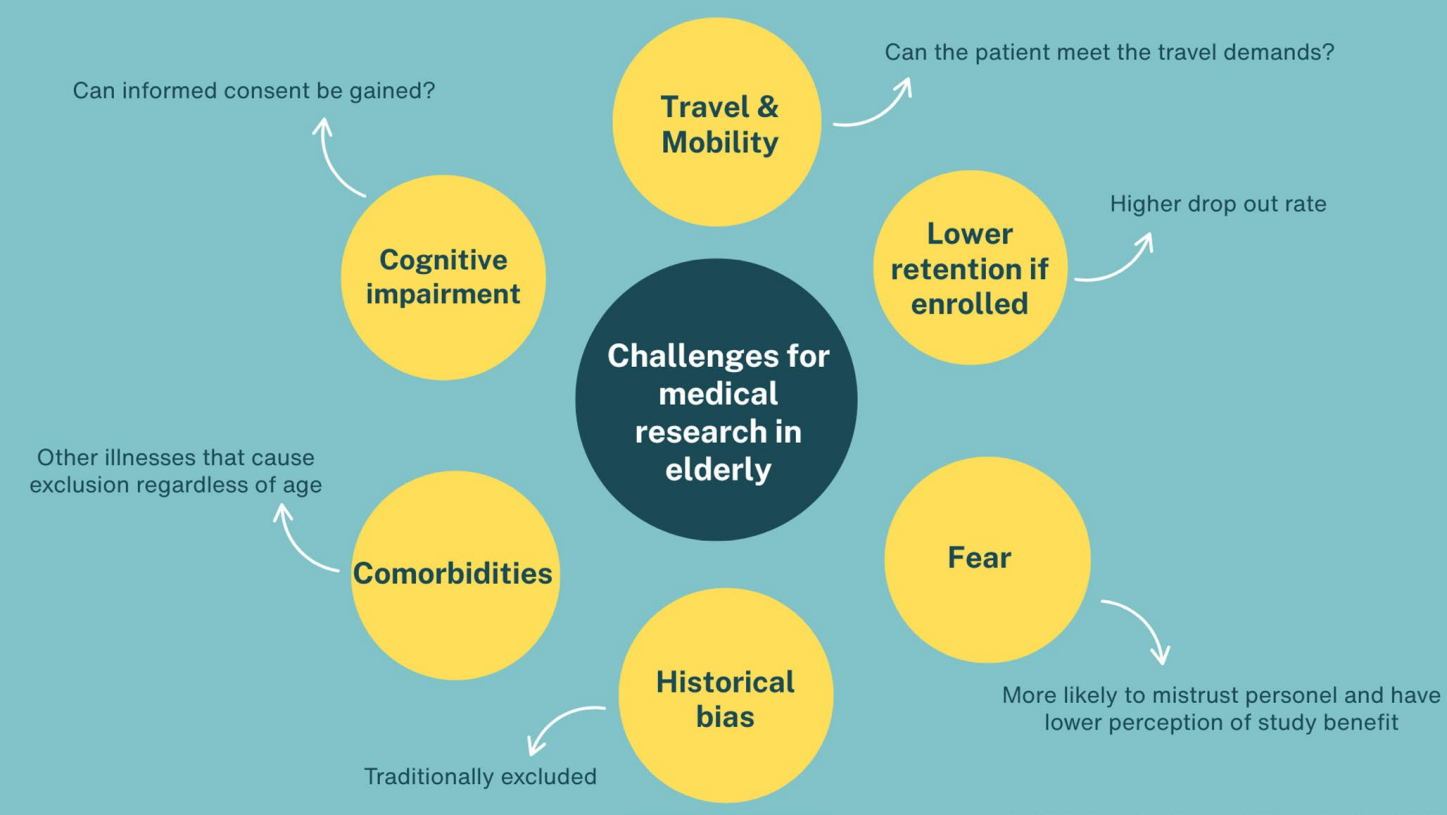
Summary of challenges for performing medical research in the elderly

There are many possible reasons why older persons are poorly represented in surgical research (Fig. 1). Firstly, the majority of clinical trials and prospective studies typically exclude elderly patients [35]. Even if eligible on age, they are more likely to have a comorbidity that is a separate exclusion criterion. Additional demands of a trial may be deemed too burdensome such as travel or additional follow up visits. Provencher et al., performed a systematic review evaluating challenges faced by the elderly in trials and cited lack of perceived benefit and distrust of research staff as commonly reported factors [36]. The higher likelihood of cognitive impairment can also raise ethical concerns regarding participation. While it is possible to gain consent for such a patient group, it is a more complex and time intensive process. From a practical perspective, the historical trend has often been to not include older persons in surgical trials as is reflected in the literature [37].

### Future Research Needs

In addition to more research on urological surgery in the elderly, these should consider incorporating outcomes that are often not routine in the index patient. While mortality and complication burden are important, there are other elements that are of great relevance for quality of life that should not be overlooked. This includes impact on functional status including mobility and home status (e.g.,* Did the surgery result in the patient being permanently relocated on a permanent basis to a nursing home? ).* Use of patient reported outcome measures (PROMs) are encouraged as these have been used to a lesser extent in elderly study samples [38].

## Conclusion

Older persons represent a complex patient group with a higher risk of complications and functional impairment associated with surgery. Careful interdisciplinary planning and implementation of geriatric assessment tools are recommended. Prospective studies are needed that both assess interventions as well as follow up those patients that adopted conservative management.

## Data Availability

No datasets were generated or analysed during the current study.

## Key References

- ******18. Graf S, Lutz J, Koneval L, Kalogirou C, Weiss SC, Bannert H, et al. Preoperative geriatric assessment to predict functional outcome after major urologic operations: Results from a multicenter study. Eur J Surg Oncol. 2024;50 [12]:108693.
  - **Evaluation of a tool to predict outcomes for elderly post surgery with focus on functional impairment**.
- ******27. Ugarte A, Bachero I, Cucchiari D, Sala M, Pereta I, Castells E, et al. Effectiveness and Safety of Postoperative Hospital at Home for Surgical Patients: A Cohort Study. Ann Surg. 2024;279 [5]:727 − 33.
  - **Original article evaluating use of intervention to support elderly undergoing surgery**.
- *****33. Ogbodo E, Talyshinskii A, Moen CA, Emiliani E, Somani BK, Tzelves L, et al. Patient consent in the modern era: Novel tools and practical considerations in urology. Current Urology. 9900:10.1097/CU9.0000000000000282
  - **Review article summarising range of consent tools now available to improve consent**.
